# Frontal Lobe Hemorrhage With Surrounding Edema and Subarachnoid Hemorrhage

**DOI:** 10.7759/cureus.31345

**Published:** 2022-11-10

**Authors:** Megan Vu, Mustafa Mohamed, Thor S Stead, Rohan Mangal, Latha Ganti

**Affiliations:** 1 Emergency Medicine, Trinity Preparatory School, Winter Park, USA; 2 Neurology, HCA Florida Osceola Hospital, Kissimmee, USA; 3 Medicine, The Warren Alpert Medical School of Brown University, Providence, USA; 4 Medicine, University of Miami Miller School of Medicine, Miami, USA; 5 Emergency Medicine, HCA Florida Ocala Hospital, Ocala, USA; 6 Emergency Medicine, Envision Physician Services, Plantation, USA; 7 Emergency Medicine, University of Central Florida College of Medicine, Orlando, USA

**Keywords:** intraventricular hemorrhage, blood pressure control, neurocritical care, hematoma expansion, intracerebral hemorrhage

## Abstract

We report the case of an 81-year-old woman who presented with a left hemineglect, a rightward gaze preference, and baseline disorientation. Her National Institutes of Health Stroke Score was 4. Her medical history was significant for dementia, osteoporosis, dyslipidemia, and a previous stroke. CT revealed a right-sided frontal lobe hemorrhage with surrounding edema and subarachnoid hemorrhage. Laboratory evaluation was significant for leukocytosis. The etiologies, clinical presentation, and diagnosis of this often devastating type of stroke are presented. While she did have a significant neurologic deficit (neglect), she was able to remain alert and protect her airway. Her hospital course consisted of observation in the ICU and blood pressure management. The case illustrates that intracerebral hemorrhage (ICH) can sometimes present indolently and does not always require surgical intervention.

## Introduction

Intracerebral hemorrhage (ICH) accounts for 15% of all strokes and 50% of stroke-related mortality, which equates to approximately 2.8 million deaths globally every year [1]. In 2010, hemorrhagic strokes accounted for nearly a third of 33 million stroke cases and had a mortality rate of just over 50% worldwide. Two major risk factors for ICH are age and anticoagulant use. The overall increase in life expectancy means a larger aging population, which translates to an increased incidence of ICH. ICH is more prevalent in older persons, and older persons are also more likely to be on an anticoagulant agent. 

Other risk factors for ICH are being part of non-white ethnicity, being male, older age, and having familial apolipoprotein syndromes [2,3]. Other risk factors also include cerebral amyloid angiopathy, uncontrolled or untreated hypertension, and drug abuse (i.e. alcohol, nicotine, and cocaine) [4]. Additional risk factors such as platelet inhibition related to COX-1 enzyme inhibitors (such as aspirin) and P2Y12 purinoceptor antagonists (such as clopidogrel, ticagrelor, prasugrel, and cangrelor) have been described [5]. 

Patients who suffer an ICH may display symptoms such as a sudden onset focal neurological deficit, followed by a decrease in the level of consciousness, which can be measured with the Glasgow Coma Scale (GCS). Other symptoms include severe headache, nausea, vomiting, convulsive and non-convulsive seizures, and elevated blood pressure [6].

## Case presentation

An 81-year-old female presented to the emergency department alongside her daughter who described the patient as being less socially interactive and refusing to engage in her usual activities. The patient's daughter reported generalized weakness and confusion. These symptoms began 2-3 days prior according to her best estimate. The daughter denied any history of trauma or head injury. She stated her mother did not have fever, headache, or other symptoms. The patient required assistance for all activities of daily living but was normally able to interact with friends and family, and engaged in several hobbies. She had occasional episodes of psychomotor agitation in the evenings and was prone to getting lost in familiar places, even inside her own home.

Her medical history was significant for dementia, osteoporosis, dyslipidemia, and a previous stroke. Her past surgical history includes a cesarean section and hysterectomy; her family history includes cancer and heart disease. She denies alcohol or recreational drug use. She was a former smoker, one pack per day for 60 years. Her temperature was 98.6 ^0^F, pulse 84 beats per minute, blood pressure 105/66 mmHg, oxygen saturation 98% on room air, and respiratory rate 18 breaths per minute.

The patient’s physical examination revealed a frail but alert lady with a left hemineglect, rightward gaze preference, and baseline disorientation. Her National Institutes of Health Stroke Score was 4. Laboratory analysis was essentially unremarkable, except for a very mildly elevated white blood cell count (Table 1).

**Table 1:** Patient's laboratory values

Laboratory Test	Reference Range	Test Result
Chemistry		
Sodium	136 - 145 mmol/L	137
Potassium	3.7 - 5.1 mmol/L	4.1
Chloride	98 - 107 mmol/L	104
Carbon Dioxide	21 - 32 mmol/L	25
Blood Urea Nitrogen	7 - 18 mg/dl	20 H
Creatinine	0.55 - 1.3 mg/dl	1.13
Glucose	74 - 106 mg/dl	104
Calcium	8.4 - 10.1 mg/dl	9.4
Total Bilirubin	0.2 - 1.5 mg/dl	0.7
Aspartate aminotransferase	10 - 37 unit/L	20
Alanine aminotransferase	12 - 78 unit/L	21
Total Alkaline Phosphatase	45 - 117 unit/L	81
Troponin I	< 54 ng/L	6
Total Protein	6.4-8.2 g/dL	8.1
Albumin	3.4 - 5.0 g/dL	3.6
Triglycerides	< 150 mg/dL	61
Cholesterol	< 200 mg/dL	167
Low Density Lipoprotein (LDL) Cholesterol	< 100 mg/dL	81
High Density Lipoprotein (HDL) Cholesterol	> 50 mg/dL	74.0
Thyroid Stimulation Hormone 3rd Generation	0.36 - 3.74 mIU/mL	1.06
Coagulation Studies		
Prothrombin time (PT)	10.0 - 12.8 seconds	12.1
International Normalized Ratio (INR)	0.8 - 1.1	1.1
Partial Thromboplastin Time (PTT)	25 - 38 seconds	29.5
Hematology		
White Blood Cell count	4.0 - 10.5 10^3 /uL	11.8 H
Red Blood Cell count	3.93 - 5.22 10^6/uL	3.95
Hemoglobin	11.2 - 15.7 g/dL	11.8
Hematocrit	34.1 - 44.9%	35.1
Platelet Count	150 - 400 10^3 / uL	300
Immature Granulocytes %	0.0 - 0.4%	0.3
Neutrophils %	34.0 - 71.1%	64.0
Lymphocytes %	19.3 - 51.7%	24.3
Monocytes %	4.7 - 12.5%	10.9
Eosinophils %	0.7 - 5.8%	0.1 L
Basophils %	0.1 - 1.2%	0.4
Nucleated RBC %	0.0 - 0.2%	0.0

**Figure 1:**
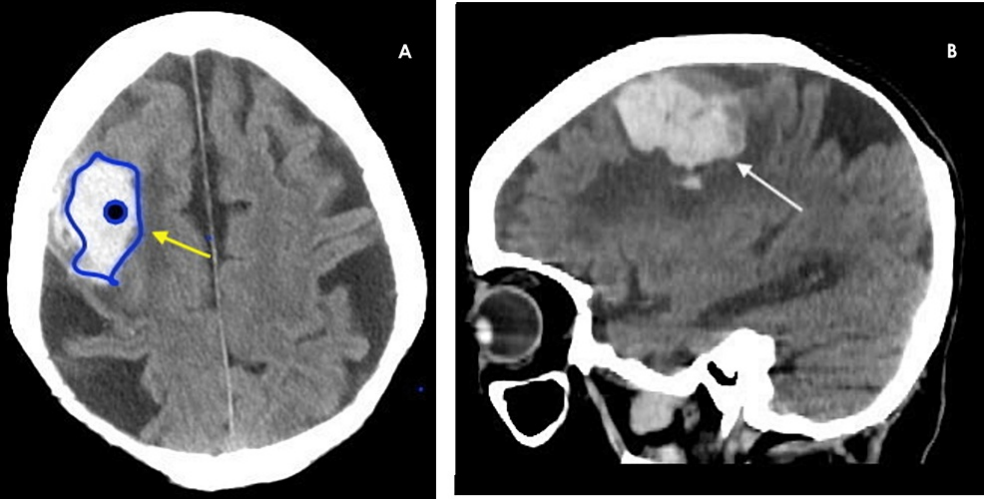
Axial (panel A) and sagittal (panel B) views of brain CT scans demonstrating right-sided intracerebral hemorrhage (ICH) located around the frontal and parietal lobes (arrow)

The patient’s brain CT demonstrated a 2.6 cm high posterior right frontal lobe hemorrhage with adjacent edema and subarachnoid blood. There was no mass effect by the hemorrhage or surrounding edema upon the right lateral ventricle (Figure 1).

The CT angiography (CTA) of her head and neck was negative for any aneurysm or arteriovenous malformation. The patient was admitted to the ICU where she had hourly neurologic checks. Systolic blood pressure was maintained between 140 and 160 mmHg. As the patient was improving spontaneously, neurosurgical intervention for craniectomy and hematoma evacuation was deferred. The patient was discharged home on day 5.

## Discussion

The location of the ICH is often helpful when trying to decipher the etiology of the hemorrhage. Subcortical and infratentorial hemorrhages are associated with hypertensive vasculopathy. Lobar and supratentorial hemorrhages, especially in patients older than age 55 are often associated with cerebral amyloid angiopathy. A review of risk factors for ICH based on the Framingham study revealed a significant association between lobar hemorrhage with hypertension, elevated systolic blood pressure, and apolipoprotein E (APOE) 4 allele homozygous status [7]. Interestingly, our patient neither had a history of hypertension nor did she have elevated blood pressure on presentation. Furthermore, her imaging demonstrates perihemorrhagic edema, which usually portends poorer outcomes. However, the patient did well despite the edema. Of note, corticosteroids were not used for our patient with edema secondary to ICH, as opposed to edema in the setting of neoplasm or cerebral infection. 

With regards to sex, there is still conflicting literature about outcomes and mortality [8]. Several studies point to higher morbidity and mortality in women with ICH secondary to the presence of intraventricular hemorrhage [9,10]. However, a 2020 European study analyzing the results of the INTERACT 1 and 2 trial noted that possible under-representation of women and data being based on single-center studies may account for uncertainty in outcomes between men and women [11]. There does, however, appear to be sex-related differences in the management of ICH. A study comparing patients who had deep infratentorial hemorrhages (locations that would be difficult for surgical intervention) found men and women to have similar rates of hydrocephalus, intraventricular hemorrhage, and ventricular shift. Even with the correction of radiographic and clinical features, men were three times more likely to have an extra-ventricular drain placed as compared to women [12]. This difference, however, was not seen when comparing hematoma evacuation rates. 

Some of the known risk factors for hematoma expansion include symptom onset to initial CT imaging, higher initial ICH volumes, concurrent antiplatelet and anticoagulant usage, and the presence of contrast extravasation on CTA, also known as the "spot sign” [13,14]. Of note, ICH volume is the strongest predictor of 30-day mortality regardless of hematoma location. When obtained within the first few hours of the first presentation, having a spot sign coincided with hematoma expansion in 77% of patients, compared to only 4%-22% without a spot sign [15]. There are other signs on imaging that portend hematoma expansion, including an irregular shape and homogenous density, the "swirl sign" (seen in panel A of our patient's brain CTA), evidence of a fluid level, a "satellite sign" (high-density starry dots around the ICH) as well as the "blend sign" (a relatively hypoattenuating area and adjacent hyperattenuating region) [16-18].

There are several surgical options for hematoma evacuation. Generally, these are considered beneficial only in patients who have signs of brainstem compression, hydrocephalus, or acute neurologic deterioration [19]. Our patient presented in a relatively stable condition, did not have major hemodynamic compromise, and was protecting her airway. In her case, observation resulted in improvement, thus no surgical intervention was undertaken.

## Conclusions

We present the case of an 81-year-old woman with ICH. The location of her ICH was lobar, thus amyloid angiopathy is a likely etiology. While she did have a significant neurologic deficit (neglect), she was able to remain alert and protect her airway. Her hospital course consisted of observation in the ICU and blood pressure management. Her symptoms improved significantly, with near resolution of her neglect. The case illustrates the use of imaging for prognostication and assessment of etiology. Despite the perihemorrhagic edema, in this case, the patient ultimately ended up doing well. The case is somewhat unusual in that the patient did not have a history of hypertension and was not hypertensive on arrival. 
